# Impact of physical fitness and biometric data on the quality of external chest compression: a randomised, crossover trial

**DOI:** 10.1186/1471-227X-11-20

**Published:** 2011-11-04

**Authors:** Sebastian G Russo, Peter Neumann, Sylvia Reinhardt, Arnd Timmermann, André Niklas, Michael Quintel, Christoph B Eich

**Affiliations:** 1 Department of Anaesthesiology, Emergency and Intensive Care Medicine, University Medical Centre Göttingen, Robert-Koch-Straße 40, 37075 Göttingen, Germany; 2 Department of Anaesthesia and Intensive Care Medicine, Evangelisches Krankenhaus Weende, Göttingen, An der Lutter 24, 37075 Göttingen, Germany; 3 Department of Orthopaedics and Trauma Surgery, University of Freiburg, Hugstetter Straße 55, 79106 Freiburg, Germany; 4 Department of Anaesthesia, HELIOS-Klinikum, Emil-von-Behring, Berlin, Walterhöfer Straße 11, 14165 Berlin, Germany; 5 Department of Anaesthesiology, Emergency and Intensive Care Medicine, Section of Sports Medicine, Georg-August University of Göttingen, Robert-Koch-Straße 40, 37075 Göttingen, Germany; 6 Department of Anaesthesia, Paediatric Intensive Care and Emergency Medicine, Children's Hospital auf der Bult, Janusz-Korczak-Allee 12, 30173 Hannover, Germany

## Abstract

**Background:**

During circulatory arrest, effective external chest compression (ECC) is a key element for patient survival. In 2005, international emergency medical organisations changed their recommended compression-ventilation ratio (CVR) from 15:2 to 30:2 to acknowledge the vital importance of ECC. We hypothesised that physical fitness, biometric data and gender can influence the quality of ECC. Furthermore, we aimed to determine objective parameters of physical fitness that can reliably predict the quality of ECC.

**Methods:**

The physical fitness of 30 male and 10 female healthcare professionals was assessed by cycling and rowing ergometry (focussing on lower and upper body, respectively). During ergometry, continuous breath-by-breath ergospirometric measurements and heart rate (HR) were recorded. All participants performed two nine-minute sequences of ECC on a manikin using CVRs of 30:2 and 15:2. We measured the compression and *de*compression depths, compression rates and assessed the participants' perception of exhaustion and comfort. The median body mass index (BMI; male 25.4 kg/m^2 ^and female 20.4 kg/m^2^) was used as the threshold for subgroup analyses of participants with higher and lower BMI.

**Results:**

HR during rowing ergometry at 75 watts (HR_75_) correlated best with the quality of ECC (*r *= -0.57, *p *< 0.05). Participants with a higher BMI and better physical fitness performed better and showed less fatigue during ECC. These results are valid for the entire cohort, as well as for the gender-based subgroups. The compressions of female participants were too shallow and more rapid (mean compression depth was 32 mm and rate was 117/min with a CVR of 30:2). For participants with a lower BMI and higher HR_75_, the compression depth decreased over time, beginning after four minutes for the 15:2 CVR and after three minutes for the 30:2 CVR. Although found to be more exhausting, a CVR of 30:2 was rated as being more comfortable.

**Conclusion:**

The quality of the ECC and fatigue can both be predicted by BMI and physical fitness. An evaluation focussing on the upper body may be a more valid predictor of ECC quality than cycling based tests. Our data strongly support the recommendation to relieve ECC providers after two minutes.

## Background

External chest compression (ECC) is a key element of cardiopulmonary resuscitation [1-4]. However, ECC is often too shallow and interrupted too frequently with resulting adverse hemodynamic effects. Furthermore, it has been demonstrated that rescuer fatigue is a serious problem affecting the quality of ECC [5-7].

The European Resuscitation Council (ERC), the American Heart Association (AHA) and other international emergency medical organisations published their guidelines for cardiopulmonary resuscitation in 2005 and 2010 [1,8-10]. As a reasonable compromise between maximised periods of uninterrupted ECC, interposed ventilations and rescuer fatigue the recommended compression-ventilation ratio (CVR) for adults was changed from 15:2 to 30:2 in 2005 [4]. Subsequent studies comparing the two CVRs gave conflicting results. While participants of one study claimed 30:2 to be more exhausting, other investigators found that the quality of ECC did not decrease with the 30:2 ratio during a 10-minute, single-rescuer scenario [11,12].

The aim of this prospective, randomised, manikin-based, cross-over study was to investigate the impact of the rescuers' physical fitness, biometry and gender on the quality of ECC using CVRs of 15:2 and 30:2. Furthermore, we aimed to determine objective parameters of physical fitness that reliably predict the quality of ECC.

## Methods

### Study participants

After obtaining the approval of the Ethics Committee of the Medical Faculty of the Georg-August-University, Göttingen, we recruited, prior to the publication of the updated guidelines for cardiopulmonary resuscitation in 2010, 30 male and 10 female volunteers with written informed consent from the Göttingen Fire Department (paramedics) and the Göttingen University Hospital (intensive-care nurses and physicians) to this exploratory study. All participants were competent in Basic Life Support (BLS) and certified Advanced Life Support (ALS) providers. No participant was taking cardiovascular or respiratory medications, had recently underone a surgical intervention, had suffered any cardiopulmonary disease or had any other cause of limited physical endurance.

### Part I: Physical fitness test

The physical fitness of all participants was evaluated by two different consecutively performed ergometric endurance tests two days before the ECC trials. First, a cycle ergometry (ERG 551, Bosch, Stuttgart, Germany) test was used following a protocol with a stepwise increase of physical strain every three minutes that started at 50 watts and was increased by 50-watt steps up to a minimum strain of 150 watts. If the participant's heart rate (HR) did not reach 100 beats per minute (bpm) at the end of the 150-watt step, a fourth step of 200 watts was added. Depending on the HR at the 50 - 150 - (or 200-) watt steps, a final maximum step was individually defined in order to reach a HR of 170 bpm. The pedal rate had to be kept constant at 50-60 revolutions/min. The workload required to reach a HR of 170 was determined as the personal watt capacity (PWC_170_), which represents a validated standard parameter for physical fitness in sport physiological investigations [13,14].

Physical exertion during ECC strains the upper more than the lower part of the body. This was addressed in a second fitness test: Each participant had to perform a three-minute ramp protocol on a rowing ergometer starting at 25 watts. Strain was increased by 25-watt steps to a minimum of 75 watts and a maximum of 125 watts. Stroke frequency had to be kept constant at between 30-40/min. As the seat of the custom-made rowing machine was not movable, all force had to be recruited from the upper part of the body.

Between the cycling and the rowing ergometry tests, all participants were allowed to recover for at least two hours. Full recovery was verified by repeated lactate measurements until two consecutive measurements had returned to the individual's baseline values.

### Ergospirometry

Ergospirometric measurements were performed with a mobile breath-by-breath ergospirometry device (Cortex Metamax 3 B™, Cortex Biophysik, Leipzig, Germany), combined with a chest-belt heart rate meter (Polar T 41™, Polar Electro, Büttelborn, Germany) during the endurance tests. We recorded respiratory rate, respiratory minute volume, oxygen consumption, carbon dioxide production and HR. All parameters were transmitted to a Windows™ based PC and recorded using MetaSoft 3.3™ software (Cortex Biophysik, Leipzig, Germany).

As PWC_170 _is a validated, standard, cycling-based parameter of physical fitness, we aimed to determine a corresponding fitness parameter evaluated during rowing exercises. Therefore, correlations for ergospirometric parameters measured during rowing with the individual PWC_170 _were calculated.

### Part II: External chest compression

Two days after part I, the participants performed two nine-minute sequences of ECC. We chose nine-minute sequences because this is approximately the average time that a first responder has to give ECC during out-of-hospital resuscitation prior to the arrival of professional healthcare providers at the scene [15,16]. Using a computer-generated randomization list, participants were randomly assigned to start ECC with a CVR of either 15:2 or 30:2, followed by a CVR of 30:2 or 15:2 in a crossover manner. Between the two ECC sequences, all participants were allowed to recover for at least 90 minutes. Full recovery was verified by repeated lactate measurements until two consecutive measurements had returned to the individual's baseline values.

**Figure 1:**
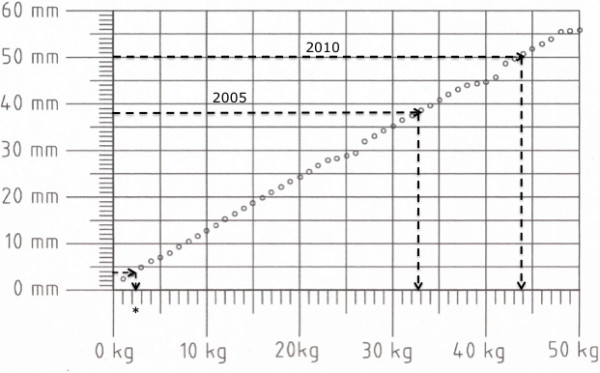
**Force-depth relationship of the manikin used in this study**. Dashed arrows indicate the corresponding force for the minimal recommended chest compression depth of 38 mm (2005 Guidelines), and 50 mm (2010 Guidelines), respectively. The star indicates the threshold used by Fried et al. to define leaning on the patient's chest [20].

ECC was performed on a standard ALS manikin (ResusciAnne™, Laerdal Medical, Stavanger, Norway) placed on the floor with a linear force-depth relationship (32.5 kg ≙ 38 mm, 44 kg ≙ 51 mm, see Figure 1). The quality of the ECC, determined by the compression depth and rate was measured and recorded by a PC-based automated skill reporting system (SkillMaster™ Reporting System, Laerdal Medical, Stavanger, Norway). In a brief pre-trial test, all participants were initially reminded to use the correct compression site (centre of the chest), rate (100 min^-1^) and depth (4-5 cm), but no corrective feedback was given during the course of the trial. The participants were advised to simulate interposed ventilations according to the CVRs of 15:2 and 30:2 by putting their mouth near the manikin's face.

As the SkillMaster™ does not provide the exact values for the *de*compression depth as well as the related compression amplitude, the databases of all the ECC cycles were processed with the MatLab^® ^programme tool identifying the minimum and maximum value of each single compression cycle (MathWorks GmbH, Ismaning, Germany).

### Subjective assessment

After the trial, all participants were asked to give their personal perceptions of the two CVRs in terms of subjective physical exhaustion and comfort.

### Statistical analyses

All statistical analyses were performed with Statistica 7.1 (Statsoft GmbH, Hamburg, Germany). Two-way ANOVA was used for statistical analysis. Tukey's HSD was used for post-hoc analyses. Linear Pearson correlations are indicated as *r*-values and were considered significant with *p*-values < 0.05. Tabular data are presented as mean values ± standard deviation or mean values ± 95% confidence interval. For illustrational reasons, the figures are displayed as mean values ± standard error of mean (SEM). *P*-values of < 0.05 were considered as significant. Detected differences of 0.1 >*p *> 0.05 were considered to represent a tendency towards significance.

## Results

All 40 participants (25 paramedics, five physicians, seven medical students and three nurses) completed the study (for biometric data, see Table 1). The highest correlation between PWC_170 _and ergospirometric parameters during rowing was found for the HR at 75 watts (HR_75_) (r = -0.85; *p *< 0.05). Note that a low HR_75 _represents a high level of physical fitness.

**Table 1:** Biometric data of all 40 participants

Parameter	Male (n = 30)	Female (n = 10)	All (n = 40)
**Age (years)**	33.86 (± 6.8)	25.3 (± 0.7)***	31.72 (± 7.0)
**Body weight (kg)**	84.43 (± 12.6)	59.30 (± 7.3) ***	78.15 (± 15.7)
**BMI (kg/m^2^)**	25.42 (± 2.6)	20.7 (± 1.7)***	24.24 (± 3.16)
**PWC_170 _(watts)**	297.7 (± 66.7)	167 (± 18.6)***	267.4 (± 80.9)
**HR_75 _(bpm)**	132.8 (± 16.8)	165 (± 15.9)***	140 (± 21.6)

Data are presented as means (± SD). BMI: body mass index; bpm: beats per minute; PWC_170_: personal watt capacity; HR_75_: heart rate at 75 Watts of rowing; ***: *p *< 0.001

We found a significant correlation between BMI, PWC_170_, HR_75 _and mean compression depth for CVRs of 15:2 and 30:2 (*r *= +0.48, +0.42 and -0.57 and *r *= +0.45, +0.40 and -0.57, respectively), indicating that participants with a higher BMI, a higher PWC_170 _and a lower HF_75 _performed deeper compressions. Analysing correlations between ergospirometric measurements and mean compression depth revealed either no significant results or results no better than those found for HR_75 _and mean compression depth.

Furthermore, in contrast to PWC_170 _and BMI, significant correlations were found between HR_75 _and the fraction of ECC with a correct compression depth (*r *= -0.55 and *r *= -0.38 for CVRs of 15:2 and 30:2, respectively) as well as HR_75 _and the fraction of ECC with too shallow compressions (*r *= +0.6 and *r *= +0.53 for CVRs of 15:2 and 30:2, respectively). Hence, in this experimental setting, HR_75 _was the best predictor for ECC performance and therefore was used as the prime variable for further correlation of physical fitness and the quality of the ECC.

Regarding the average values of all participants over nine minutes of ECC, the no-flow time for 30:2 was significantly less than for 15:2. All ECC data comparing 15:2 and 30:2 are presented in Table 2. All participants *de*compressed the chest incompletely during ECC (Table 2). Therefore, for both CVRs the compression *amplitude *was significantly lower for male and female participants as compared to the compression depth (data not shown, *p *< 0.001; t-test for paired data). As the *de*compression depth, however, did not change over the nine minutes of ECC, further analyses were focused on both the compression depth and compression rate.

**Table 2:** Values of external chest compression variables for the participants as means over a nine-minute period, for all participants and differentiated by gender.

Parameter	15:2	30:2	15:2	15:2	30:2	30:2
	
	All (n = 40)	All (n = 40)	Male (n = 30)	Female (n = 10)	Male (n = 30)	Female (n = 10)
**Compression rate (n/min)**	**110 **(104/116)	**108 **(102/115)	**107 **(100/114)	**120 **(109/131)*	**106 **(99/112)	**117 **(102/133)
**Compression depth (mm)**	**38.3 **(35.6/41.0)	**37.4 **(34.2/40.6)	**40 **(37/43)	**32 **(27/38)**	**39 **(35/43)	**32 **(26/37)**
**Correct compression depth (%)**	**32 **(21/43)	**33 **(21/45)	**36 **(22/48)	**23 **(-2/48)	**38 **(22/52)	**19 **(-1/40)
**Too shallow (%)**	**55 **(42/68)	**55 **(40/69)	**48 **(33/63)	**77 **(50/102)**	**46 **(25/63)	**80 **(60/101)*
**Too deep (%)**	**15 **(4/25)	**12 **(3/21)	**19 **(6/32)	**0.5 **(0/1.5)	**16 **(5/28)	**0**
**Decompression depth (mm)**	**2.3 **(1.8/2.9)	**2.2 **(1.8/2.7)	**2.5 **(1.8/3.2)	**1.7 **(1.2/2.2)	**2.4 **(1.9/3.0)	**1.6 **(1.1/2.1)
**No-flow time (sec)**	**273 **(262/283)	**188 **(179/197)***	**272 **(260/285)	**273 **(252/295)	**187 **(176/198)	**190 **(172/208)

Data are presented as means (± 95% confidence interval). *P*-values refer to the comparison between female and male participants. *: *p *< 0.1; **: *p *< 0.05; ***: *p *< 0.001 (note: comparing 15:2 vs. 30:2).

**Figure 2:**
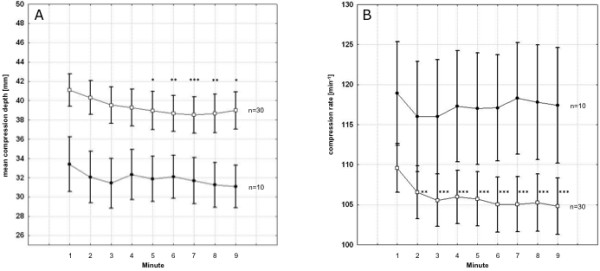
**Minute-to-minute compression depth (A) and rate (B) during external chest compression performed by male (n = 30) and female (n = 10) participants**. **A: **Compression depth, male vs. female: *p *= 0.04; **B: **Compression rate, male vs. female: *p *= 0.1. Squares = male participants; black circles = female participants. Asterisks indicate significant differences comparing the first minute with the subsequent minutes of compression within each group. *: *p *< 0.1; **: *p *< 0.05; ***: *p *< 0.01. Data are presented as mean ± standard error of mean.

Minute-to-minute analysis of all participants showed a significant decrease in compression depth starting from minute four (94.8% of minute 1) for 15:2 (*p *< 0.05) and from minute three (95.3% of minute 1) for 30:2 (*p *< 0.05). Furthermore, female participants compressed more rapidly (*p *= 0.1) and significantly more shallowly (*p *= 0.04) than male participants (Figures 2A and 2B).

### Separation based on biometric data

For the entire cohort, we found a significant correlation between gender and BMI as well as gender and HR_75_. Furthermore, a significant correlation between BMI and HR_75 _was seen (*r *= -0.58), potentially indicating BMI as an epiphenomenon of good physical fitness due to an increased muscle mass. Finally, significant differences in the quality of ECC were found between female and male participants with regards to compression depth and rate (see Table 2). We therefore analysed female and male participants separately. In addition, male and female participants were differentiated into groups with higher and lower values of BMI and HR_75_. The calculated median of each variable was set as the threshold between the high and low groups. Thus, half of the cohort (15 males and five females) represented the highs and the lows. The median values were as follows: For male participants BMI = 25.4 kg/m^2 ^and HR_75 _= 130.5 bpm; for female participants BMI = 20.4 kg/m^2 ^and HR_75 _= 167.0 bpm. In the following, for male participants lower BMI refers to participants with a BMI below 25.4 kg/m^2^; a higher BMI refers to participants with a BMI above 25.4 kg/m^2^. For females, a lower BMI refers to participants with a BMI below 20.4 kg/m^2 ^and a higher BMI to participants with a BMI above 20.4 kg/m^2^.

**Figure 3:**
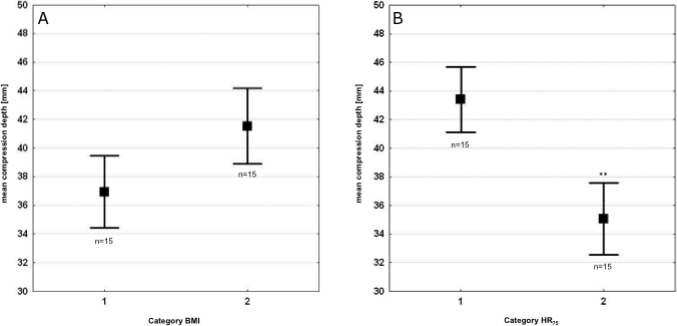
**Average compression depth of nine minutes of external chest compression performed by male participants**. **A: **categorised for lower and higher Body Mass Index (BMI); **B: **categorised for lower and higher heart rate at 75-watt rowing (HR_75_). Category BMI: 1 = participants with lower BMI; 2 = participants with higher BMI; Category HR_75_: 1 = participants with lower HR_75_; 2 = participants with higher HR_75_; **: *p *< 0.05. Data are presented as mean ± standard error of mean. For the definition of lower and higher HR_75 _and BMI, respectively, please refer to the main text.

We found no significant correlation between BMI and HR_75 _(*r *= 0.33) for male participants. The influence of BMI on the average compression depth provided by male participants failed to reach the significance level (Table 3 and Figure 3A). However, HR_75 _significantly determined the average compression depth of both CVRs (*p *= 0.02; Table 3 and Figure 3B).

**Table 3:** Mean values over a nine-minute period of external chest compression variables for male participants, differentiated for males with higher (n = 15) and lower (n = 15) BMI and HR_75 _values, respectively.

Parameter	15:2 lower BMI	15:2 higher BMI	15:2 lower HR_75_	15:2 higher HR_75_	30:2 lower BMI	30:2 higher BMI	30:2 lower HR_75_	30:2 higher HR_75_
**Compression rate (n/min)**	111 (100/123)	103 (92/113)	105 (94/116)	109 (98/120)	111 (100/122)	100 (92/109)*	101 (91/111)	111 (101/120)
**Compression depth (mm)**	37.9 (33/42)	42 (38/46)	43.1 (39/47)	37.3 (33/42)**	36.9 (31/42)	41.5 (35/47)	43.4 (38/48)	35.1 (30/41)**
**Correct depth (%)**	26.6 (10/43)	44 (24/65)	55.7 (37/74)	15.4 (4/27)***	39.0 (15/63)	36.1 (14/58)	50.8 (28/74)	24.4 (3/45)*
**Too shallow (%)**	61.6 (40/83)	34.7 (13/57)	25.3 (7/43)	71.0 (51/91)***	52.5 (25/80)	39.6 (14/65)	26.2 (4/49)	65.8 (40/91)**
**Too deep (%)**	11.7 (-5/28)	27 (5/49)	25.2 (5/46)	13.6 (-5/33)	8.4 (-4/20)	24.3 (3/45)	23.0 (3/43)	9.8 (-5/24)
**Decompression depth (mm)**	2.0 (1.3/2.7)	3.0 (1.7/4.3)	2.9 (1.9/3.9)	2.1 (1.0/3.2)	2.2 (1.4/3)	2.7 (1.8/3.6)	2.9 (2.0/3.7)	2.0 (1.2/2.7)

Data are presented as means (± 95% confidence interval). *P*-values refer to the comparison between lower vs. higher Body Mass Index (BMI) and lower vs. higher heart rate at 75 watts of rowing (HR_75_). *: *p *< 0.1; **: *p *< 0.05; ***: *p *< 0.001. For the definition of lower and higher HR_75 _and BMI please refer to the main text.

Analysing the minute-to-minute compressions, the ECC depths performed by male participants with a lower BMI declined significantly over the nine-minute sequence starting at minute six for 15:2 (*p *< 0.05) and at minute five for 30:2 (*p *< 0.05).

**Figure 4:**
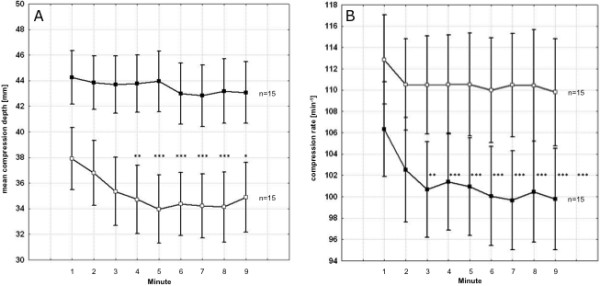
**Minute-to-minute results of external chest compression performed by male participants for 30:2 in correlation to physical fitness determined by the heart rate at 75 watts of rowing (HR_75_)**. **A: **compression depth, lower vs. higher HR_75_: *p *< 0.001; **B: **compression rate, lower vs. higher HR_75_: *p *< 0.001. Black squares = low HR_75_; white squares = high HR_75_. Asterisks indicate significant differences comparing the first minute with the subsequent minutes of compression within each group. *: *p *< 0.1; **: *p *< 0.05; ***: *p *< 0.01. Data are presented as mean ± standard error of mean. For the definition of lower and higher HR_75 _please refer to the main text.

Compression depth did not decrease when performed by male participants with lower HR_75 _in both CVRs. For 15:2, compression depth performed by male participants with higher HR_75 _started to decline significantly from minute six (93% of minute one, *p *< 0.01) and from minute four (95.0%, *p *< 0.01) for 30:2 (Figure 4A).

Male participants with a lower BMI and higher HR_75 _compressed significantly more rapidly than male participants with a higher BMI and lower HR_75_, particularly at the beginning of the nine-minute sequence. The decrease of compression frequency over time was significant when performed by male participants with a lower HR_75 _for both 15:2 and 30:2 (*p *< 0.05 and < 0.01, respectively). Consequently, the compression frequencies approximate the recommended rate in 2005 with 100 per minute for male participants with a lower HR_75 _(Figure 4B).

No correlation between BMI and HR_75 _was observed in female participants (*r *= 0.05). In the minute-to-minute analyses, the initial compression depths did not differ, independently whether performed by female participants with lower or higher BMI. However, the decrease of the compression depth over time was significant when performed by female participants with a lower BMI for both 15:2 and 30:2 (*p *< 0.01 and < 0.05, respectively).

Compression depths tended to be higher in females with lower HR_75 _for both CVRs. These differences failed to reach the significance level. The compression frequencies remained high and did not decline over the nine minutes for either CVR, as well as those performed by female participants with low and high HR_75 _or BMI.

### Subjective assessment

The majority (68%) of all participants assessed 30:2 to be more exhausting. Both CVRs were assessed as equally exhausting by 25%. Nevertheless, 55% experienced 30:2 to be the more comfortable regimen (versus 35% for 15:2).

## Discussion

We investigated the impact of physical fitness, BMI and gender of the provider on the quality of ECC when performing CVRs of 15:2 and 30:2. Our main findings are as follows: 1) good physical fitness and a higher BMI (in this study above 25.4 kg/m^2^) correlate positively and independently of gender with the quality of ECC (primarily defined by correct compression depth and rate); 2) female participants performed ECC that was too shallow and more rapid as compared to male participants; 3) compression depth decreased over time among less fit participants and participants with a lower BMI; 4) a CVR of 30:2 was rated to be more exhausting but also more comfortable; 5) physical fitness tests focusing on the upper body of the health care provider may be a reliable tool to predict the quality of ECC.

Our study confirmed the calculation that a CVR of 30:2 results in a higher number of compressions and a consequential reduction in no-flow time as compared to 15:2 [12,17]. Other ECC data, such as compression, decompression depths and compression amplitude, did not statistically differ between the two CVRs, which confirms previous data [11]. Nevertheless, rescuer fatigue, reflected by a decrease of compression depth over time, occurs at an earlier stage and is more pronounced for 30:2 compared to 15:2.

Physically fit rescuers as well as rescuers with a higher BMI showed better ECC performance and significantly less fatigue. More importantly, a higher BMI in this study was not an epiphenomenon of higher physical fitness due to increased muscle mass. It seems important to point out that the study participants with higher BMIs decompressed the chest to a lesser extent than those with lower BMIs, independently of gender. Although these differences are not statistically significant, participants with higher BMIs should be reminded to avoid leaning on the patients' chest in order to fully decompress the chest, and thus provide optimal circulatory support as highlighted in the updated 2010 ERC Guidelines [1]. Leaning on the patient's chest seems to be a common occurrence [18], and several authors recently addressed this adverse phenomenon [19,20]. In a clinical observational study, Fried et al. defined leaning as the presence of force above 2.5 kg at the point of minimum chest compression depth (*de*compression depth) and found a wide range of leaning during chest compressions [20]. In contrast, in this manikin-based study we found that *all *our participants failed to let the chest recoil completely. With the MatLab™ analyses, we might have been able to detect leaning in a more sensitive manner. However, the differences between clinical and manikin-based studies need to be acknowledged and, in addition, different definitions and thresholds for leaning may hinder study comparisons and assessments of clinical importance [20-22]. Indeed, in our study, 2.5 kg would represent a compression depth of approximately four millimetres and, even in participants with a higher BMI, we rarely found a *de*compression depth above this threshold.

Our data support previous results regarding the influence of physical fitness on ECC performance [6,7,23]. However, in contrast to Lucia et al., we evaluated two fitness parameters focussing on both lower (PWC_170_) and upper body parts (HR_75_). As we found a higher correlation between compression depth and HR_75 _as compared to compression depth and PWC_170_, our findings may suggest that fitness tests focussing on the upper body (e.g., rowing ergometry), rather than the lower body (e.g., cycle ergometry tests [7]), or even self-reporting questionnaires on physical fitness [24], may be more helpful for predicting the quality of ECC.

Even though previous studies included male and female participants [6,11,25-27], few studies distinguished between them [23-25]. Our findings support those from Ashton et al. and Paberdy et al., both suggesting an impact of gender on a satisfactory performance of ECC [6,26]. Furthermore, our data confirm results by Paberdy et al., who showed a significantly higher compression rate by female providers, was well as recently published data by Hansen et al., who demonstrated that the quality of ECC performed by females was lower than that by male participants [23]. However, our female participants had a significantly lower BMI. As we found that participants with a lower BMI tended to perform shallower and more rapid compressions than those with a higher BMIs, different BMIs may at least partly explain the gender-related differences. This gives credit to a previous assumption that rescuer fatigue during ECC may be underestimated by lighter rescuers [6]. As the percentage of female paramedics is increasing in many emergency medical services, female rescuers should take special care to perform sufficient ECC.

It is a matter of fact that any kind of ECC is more favourable for patient outcome than no ECC at all. However, the updated ERC guidelines from 2010 dictate deeper compressions than the 2005 guidelines (see Figure 1) [2,4]. Given the overall risk of potentially low-quality ECC [28,29] and the significant influence of physical fitness and biometric data on the quality of ECC, our data emphasise the necessity of physically well-trained healthcare providers, frequent alternation of rescuers during ECC [2], the use of feed-back devices [30] and, particularly important, addressing the phenomenon of rescuer fatigue during training in CPR.

We found a significant decrease of ECC depth over time, and that this was more pronounced in less fit and lighter providers, and occurred at an earlier stage for the 30:2 CVR than for 15:2. This stands in contrast to data presented by Bjorshol et al. [12] and Jantti et al. [27] but was in accordance with other available data [5,6]. These inconsistencies may be explained by the different time frames analysed (one-minute frames in this study vs. two-minute frames in study [12]) or by the lack of subgroup analyses. Indeed, the reduction of ECC depth over time in Jantti's study failed to reach significance level but showed an analogous tendency (*p *= 0.079).

According to the participants' subjective perception, 30:2 was more exhausting but was also rated as more comfortable. This confirms the subjective evaluation of participants by Deschilder et al. [11]. Our findings may be attributed to the frequency of interruption of ECC by interposed ventilations.

### The model

There is an ongoing discussion as to whether standard resuscitation manikins and manikin-based scenarios may sufficiently reflect clinical reality [31]. First, in contrast to a linear relation in manikins, there is a rather non-linear relation between compression depth and force to be applied to the human chest [32]. Secondly, rescuers, although physically capable of performing effective ECC, may refrain from performing correct ECC because of fear of injuring the patient, particularly when the patient's chest is rather stiff. Nevertheless, controlled investigations on the technical quality of ECC related to variable conditions are usually performed on resuscitation manikins [6,12,33,34]. Their mechanical properties facilitate training and assessment of the characteristics of chest compression, decompression and rate [35]. Factors that do have an impact on the quality of ECC, such as BMI, physical fitness and gender remain the same irrespective of the depth-force relation. Furthermore, the average force needed to compress a patient's elastic chest to the recommended minimum depth of 38 mm has been determined to be 27.5 ± 13.6 kg [32]. The force-depth relation of the manikin used in this study (32.5 kg for 38 mm) accurately reflects this clinical reality. Moreover, the stressful setting of a clinical resuscitation could distract healthcare providers from focusing their attention on the correct performance of ECC, hence physical fitness and biometric parameters may unconsciously influence the quality of ECC even more.

### Limitations

We are aware of the limited number of female participants, which may have prevented us from gaining significance levels of *p *< 0.05 in several tests and may have caused a bias in evaluating the entire cohort. However, gender-based analyses revealed parallel results for male and female rescuers. As our cohort consisted of professional healthcare providers, our results may differ from those found among laypersons. Furthermore, our participants only imitated rescue breaths, and fatigue during actual CPR with correctly provided rescue breaths or during continuous ECC (e.g. in the context of hands-only CPR or during continuous ECC with a secured airway) might therefore be different, Moreover, our study was performed prior to the publication of the updated 2010 guidelines, and the correct compression depth was therefore defined as 38-51 mm according to the 2005 guidelines.

## Conclusion

Physical fitness and higher BMI (in this study above 25.4 kg/m^2^) correlated positively with a sustained high quality of ECC. A physical fitness test that incorporates the upper part of the body, such as rowing ergometry, facilitates the prediction of the quality of ECC. The initial quality was not significantly different between the two CVRs, but rescuer fatigue occurred earlier for 30:2 than for 15:2. Female participants tended to compress too shallowly and too rapidly. Our data on rescuer fatigue strongly supported the recommendation to relieve the provider every two minutes during ECC as well as the use of feedback devices to assure high-quality chest compressions.

## Competing interests

The authors declare that they have no competing interests.

## Authors' contributions

SGR conceived and designed the study, made substantial contribution to data acquisition, performed statistical analyses and data interpretation, drafted the manuscript. PN made substantial contributions to data analyses. SR substantially recruited participants and carried out data acquisition. AT helped to draft the manuscript. AN made substantial contribution to data acquisition regarding physical fitness parameters. MQ helped to draft the manuscript. CE drafted the manuscript and made substantial contribution to data interpretation. All authors read and approved the final manuscript.

## Pre-publication history

The pre-publication history for this paper can be accessed here:

http://www.biomedcentral.com/1471-227X/11/20/prepub
